# Pulmonary Cryptococcosis: comparison of Cryptococcal antigen detection and radiography in Immunocompetent and Immunocompromised patients

**DOI:** 10.1186/s12879-020-4818-1

**Published:** 2020-01-30

**Authors:** Jingqi Min, Kunlun Huang, Chanmei Shi, Laifu Li, Fuye Li, Tao Zhu, Huojin Deng

**Affiliations:** 1 Department of Pulmonary and Critical Care Medicine, Zhujiang Hospital, Southern Medical University, Guangzhou, 510280 China; 2 Department of Pulmonary and Critical Care Medicine, Second Affiliated Hospital, Chongqing Medical University, Chongqing, China

**Keywords:** Cryptococcosis, Immune, Serum cryptococcal antigen (CrAg)

## Abstract

**Background:**

We compared the cryptococcal antigen detection and imaging findings between immunocompetent and immunocompromised patients in whom pulmonary cryptococcosis had been diagnosed. The aim of our study was to determine whether the patient’s immune status and radiography affect the detection of cryptococcal antigen.

**Methods:**

According to whether they took immunosuppressive drugs or not, seventy and eight adult patients with pulmonary cryptococcosis were divided into two groups: the immunocompetent group and the immunocompromised group. According to the detection of CrAg, each group was divided into the CrAg+ group and the CrAg- group. Then, clinical records, laboratory examinations and computed tomography findings were collected and analyzed.

**Results:**

No difference was found in baseline characteristics, clinical symptoms, and laboratory investigations. By comparing CrAg detection in these two groups, it was found that the number of CrAg+ cases in the immunocompetent group was more than that in the immunocompromised group. And in the immunocompetent group, diffuse lesions were more common in CrAg+ group and limited lesions were more frequently observed in CrAg- group.

**Conclusions:**

The patient’s immune status and radiography would affect the detection of cryptococcal antigen. And serum CrAg could be a useful tool for the diagnosis of pulmonary cryptococcosis in immunocompetent patients with extensive lung involvement.

## Background

Pulmonary cryptococcosis caused by *Cryptococcus neoformans C. Cryptococcus complexes* is very common in immunocompromised individuals, and it has become an emerging disease in immunocompetent ones [1–3]. Some reports have shown that pulmonary cryptococcosis occurs more frequently in immunocompetent patients than in immunocompromised ones [4, 5]. Compared with the current high incidence, the diagnosis of pulmonary cryptococcosis is still difficult because the clinical manifestations and imaging of pulmonary cryptococcosis have no obvious characteristics compared with other pulmonary diseases [6–8]. At present, diagnostic therapy and lung biopsy are mostly used to help diagnosis in China. But both methods have their drawbacks. Diagnostic using antifungal drugs have a long diagnosis cycle and side effects, and lung biopsy also has some indications, and it is invasive, difficult and complex to operate. Therefore, it is necessary to find a non-invasive and reliable method with few side effects. Some research [9–12] have shown that serum CrAg is a useful diagnostic methods. However, Serum CrAg detection negative is common among patients with Pulmonary cryptococcosis. Recent study [13] has shown that serum CrAg is a useful diagnostic tool for pulmonary cryptococcosis in patients with extensive lung invasion. Based on that, we speculate that the radiological manifestations may affect the detection of serum CrAg. In addition, in a large number of clinical observations, we found that the immune status will also have an impact on their detection. (because the incidence of AIDS in China is relatively low, so it will not be discussed here.) Considering the above information, we want to figure out whether the pulmonary radiography and immune status could affect the CrAg detection.

## Methods

### Study subjects

**Fig. 1:**
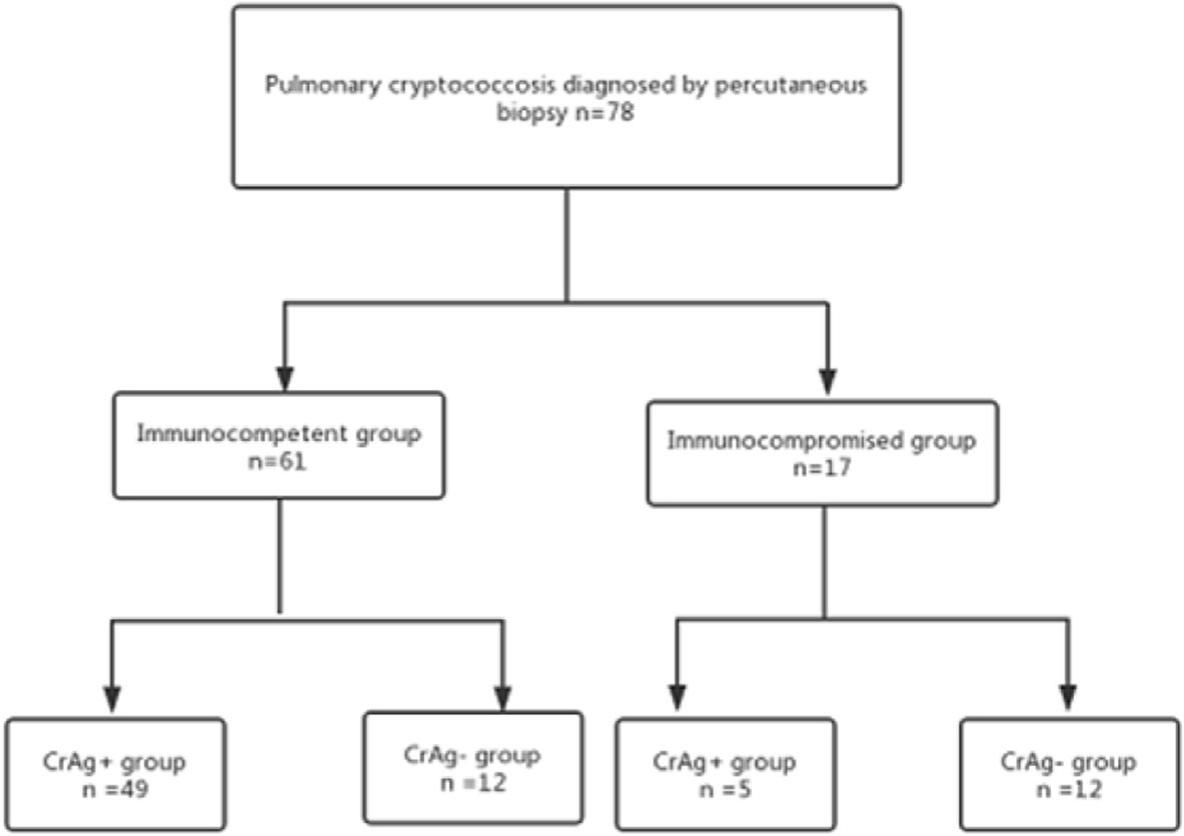
Flow diagram of the study patient selection

We retrospectively examined the hospitalized patients with pulmonary cryptococcosis between January 2012 and March 2019 in Zhujiang Hospital of Southern Medical University (Guangdong, China), Hospital of Southern Medical University (Guangdong, China) and Guangzhou People’s Hospital (Guangdong, China). All patients’ diagnoses were confirmed by percutaneous transthoracic needle biopsy (PTNB) or postoperative biopsy, and diagnosed by histopathological observations. Then, the data and information were collected: (1) demographic features and past medical history; (2) clinical features and symptoms; (3) laboratory tests, including white blood cell (WBC) counts, neutrophils counts, lymphocytes counts, concentrations of hemoglobin (Hb),platelet counts (PLT); (4) serum CrAg was detected by lateral flow assay (LFA) (IMMY, Norman, OK, USA); (5) chest radiological findings [Fig. 1]. Seventy-eight patients with pulmonary cryptococcosis were divided into two groups: the immunocompetent group and the immunocompromised group, according to whether they took immunosuppressive drugs or not. According to the detection of CrAg, each group was divided into the CrAg+ group and the CrAg- group. Then, according to Tao Zhu’s research [13], we define diffuse lesions as multiple lobes of the lung, multiple lesions of a single lobe, or lesions of a single lobe larger than 3 cm in diameter. Limited lesions are defined as lesions with a diameter of less than 3 cm in a single lobe. This study was approved by research Southern Pearl River Hospital Ethics Committee Medical University (No. 2016 HXNK 007), and all patients signed informed consents.

### Statistical analysis

Statistical analyses were performed with SPSS software, version 20.0. Continuous data are presented as the mean ± standard deviation or range. Chi-square test was used to analyze categorical variable. The independent t-test was obtained to analyze continuous variable. *P* < 0.01 was considered statistically significant.

## Results

### Demographics and clinical data

Patient demographic and clinical information is summarized in Table 1. Our patients ranged in age from 15 to 81 years (mean age, 44.44 years). Forty-nine patients were male and 29 were female. Sixty-one patients had no immunodeficiency disorders, and 17 patients had received immunosuppressive therapy for a disease, 15 renal transplant patients received immunosuppressive therapy with cyclosporine, corticosteroids and mycophenolate. Two patients with acute leukemia received VP regimen (vincristine + prednisone) as chemotherapy regimen. Cough was the most common presenting symptom, occurring in 44 immunocompetent patients (72.13%) and 15 in immunocompromised patients (88.24%), followed by expectoration (55.74% vs 58.82%), chest pain (22.95% vs 23.53%), and fever (13.11% vs 17.65%). Nineteen people were asymptomatic,17 of whom had no immunodeficiency disease. There was no significant difference in the levels of WBC, neutrophils, lymphocytes, Hb and PLT between two groups (Table 2).

**Table 1:** Medical history of patients with pulmonary cryptococcosis

Characteristics	Immunocompetent group	Immunocompromised group	P
n	61	17	
Male(%)	38(62.0)	11(64.0)	0.86^c^
Age	44.82	43.06	0.65^t^
Asymptomatic (%)	17(27.87)	2(11.76)	0.29^c^
Cough(%)	44(72.13)	15(88.24)	0.29^c^
expectoration (%)	34(55.74)	10(58.82)	0.82^c^
Chest pain (%)	14(22.95)	4(23.53)	1.00^c^
Fever (%)	8(13.11)	3(17.65)	0.94^c^
Underlying diseases (%)	2(0.03)	17(100)	
Hypertension	2	0	
Kidney transplantation	0	15	
Leukemia	0	2	

^c^Chi-square test; ^t^t-test

**Table 2:** Laboratory investigations of patients with pulmonary cryptococcosis

Characteristics	Immunocompetent group	Immunocompromised group	P
WBC (10^9^/L)	7.27 ± 1.96	7.93 ± 2.57	0.26^t^
Neutrophils	4.87 ± 1.92	5.83 ± 1.94	0.07^t^
Lymphocytes	1.89 ± 0.57	1.75 ± 0.38	0.66^t^
Hb(g/L)	131.00 ± 17.87	121.70 ± 23.47	0.08^t^
PLT (10^9^/L)	291.98 ± 107.67	258.41 ± 116.67	0.26^t^

^c^Chi-square test; ^t^t-test. *WBC* White blood cell; *Hb* Hemoglobin

### Serum cryptococcal antigen and immune status

By comparing the results of cryptococcal antigen test in two groups, we found that the number of CrAg+ cases in the immunocompetent group was more than that in the immunocompromised group (49 vs 5), which was a significant statistical difference between the two groups (*P* < 0.01). (Table 3).

**Table 3:** Comparison of Cryptococcal Antigen Detection in the Immunocompetent and Immunocompromised group

CrAg expression	Immunocompetent group	Immunocompromised group	P
CrAg+	49	5	0.0001^a^
CrAg-	12	12	

^a^significant at 99% CI; *CrAg* Cryptococcal antigen

### Chest radiology

**Fig. 2:**
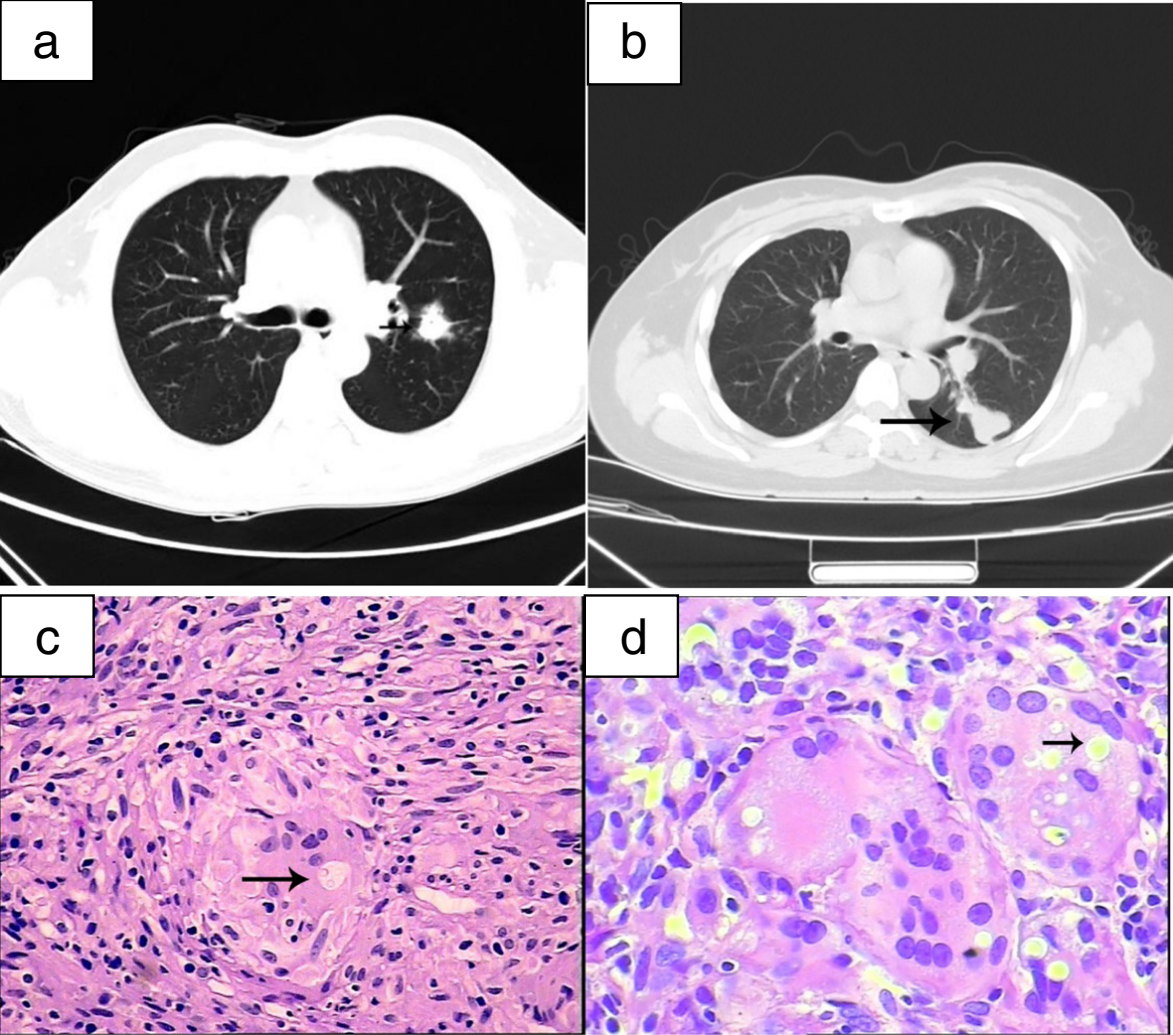
The chest radiological findings and biopsy results in patients with pulmonary cryptococcosis. (**a**) lesion (the diameter < 3 cm) in a single lobe (limited extent lesion). (**b**) lesions in multiple lobes (diffuse extent); Lesion (black arrows). Biopsy results (**c-d),** Cryptococcus sp. yeasts (black arrows)

Table 4 showed that diffuse extent lesion was more common in CrAg+ group and limited extent lesion was more frequently observed in CrAg- group among the immunocompetent group (47 vs 7)(P < 0.01). On the contrary, in the immunocompromised group, more diffuse lesions were found in the CrAg- group than in the CrAg+ group (10 vs 4)(*P* > 0.01), as shown in Table 5. Chest radiology and biopsy results in patients with pulmonary cryptococcosis were shown in Fig. 2.

**Table 4:** Comparison of Cryptococcal Antigen Detection and Radiography in the Immunocompetent group

Characteristics	CrAg+	CrAg-	P
Diffuse extent lesion	47	7	0.002^a^
Limited extent lesion	2	5	

^a^significant at 99% CI; *CrAg* Cryptococcal antigen

**Table 5:** Comparison of Cryptococcal Antigen Detection and Radiography in the Immunocompromised group

Characteristics	CrAg+	CrAg-	P
Diffuse extent lesion	4	10	1^a^
Limited extent lesion	1	2	

^a^significant at 99% CI; *CrAg* Cryptococcal antigen

### Treatment

There were 12 patients who received surgical resection, 5 patients who received surgery combined with antifungal drugs, 58 patients who received Antifungal monotherapy (56 cases were treated with fluconazole, 2 cases were treated with amphotericin), and 3 patients who were confirmed by biopsy were discharged from the hospital without treatment. (Table 6).

**Table 6:** Comparison of treatment in the Immunocompetent and Immunocompromised group

Characteristics	N	Operation	Fluconazole monotherapy	Operation combined with fluconazole	Amphotericin monotherapy	Other
Immunocompetent group	61	10	3	48	0	0
Immunocompromised group	17	2	2	8	2	3

### Prognosis

The follow-up data of 23 patients were complete. In the immunocompetent group, all lesions were removed by operation in four patients and no recurrence was found in follow-up for several months. And 15 patients were treated with fluconazole alone. After treatment, it was found that all lesions were absorbed. In the immunocompromised group, 2 patients were treated with surgery combined with fluconazole, 1 patient was treated with fluconazole initially, and then was treated with amphotericin and sequential voriconazole. Lesions were cleared in all 3 patients. And all of them were limited extent lesion.

## Discussion

In this study, a retrospective research of 78 patients with pulmonary cryptococcosis was conducted to investigate whether the patient’s immune status and radiography affect the detection of cryptococcal antigen. Firstly, according to the patient’s immune status, we divided these pulmonary cryptococcosis patients diagnosed by lung biopsy into the immunocompetent group and the immunocompromised group. We found the number of CrAg+ cases in the immunocompetent group was more than that in the immunocompromised group. This may indicate that the positive detection of serum cryptococcal antigen in immunocompetent individuals is more likely to represent the infection of pulmonary cryptococcosis. Moreover, in the immunocompetent group, diffuse lesions were more common in CrAg+ group and limited lesions were more frequently observed in CrAg- group. It suggests that the possibility of pulmonary cryptococcal infection is high, when immunocompetent patients with diffuse lung lesions have a positive serum cryptococcal antigen test. It has been reported that the detection of serum cryptococcal antigen is often negative in patients who have isolated pulmonary, and the presence of a positive serum cryptococcal antigen means the high possibility of deep tissue infiltration and disseminated diseases, [14–16]. But the result in the immunocompromised group was contrary (*p* > 0.01), which may be related to the small sample size. The radiological findings of patients with pulmonary cryptococcosis are non-specific, and depend on the immune status of patients. In immunocompetent patients, solitary or multiple pulmonary nodules are common, while in immunocompromised patients, cavities often appear [15, 17]. Our findings in immunocompetent individuals have expanded the value of serum CrAg in the diagnosis of diffuse lesion in immunocompetent patients, supporting the results of previous studies that the most common CT findings of pulmonary cryptococcosis was multiple pulmonary nodules/masses of different diameters [18–21].

Similar to previous reports, our study also found that the most common symptoms of pulmonary cryptococcosis patients included cough and sputum, [5, 22–24] with no significant specificity(Table 1). Our laboratory examinations, including leucocyte, neutrophil, lymphocyte, Hb and PLT levels, showed no significant difference between immunocompetent and immunodeficient groups (Table 2). This may confirm that cryptococcosis can inhibit the aggregation of pulmonary neutrophils and the production of inflammatory cytokines in immunocompetent host, thus affecting the immune function of healthy subjects [23, 25].

In conclusion, patient’s immune status and CT manifestations would affect the detection of serum cryptococcal antigen. Serum CrAg could become a useful tool for the diagnosis of pulmonary cryptococcosis in immunocompetent patients with extensive lung involvement.

### Future work

Of course, there are some limitations. First of all, this is a retrospective study. We collected a relatively small number of cases, so we will expand the sample size and a series of cohort studies will be conducted next. And as pulmonary physicians, our research is limited to pulmonary cryptococcosis. In the future, we will strengthen cooperation with other departments and hospitals to deepen our understanding of cryptococcal and serum cryptococcal antigen detection. Finally, because of the incomplete data, we have not classified the pathogenic bacteria of cryptococcosis. From now on, we will continue to study whether the infection of specific Cryptococcus is related to the immune status of patients, CT findings and cryptococcal antigen detection.

## Data Availability

The data that support the findings of this study are available from the corresponding author upon reasonable request.

## Abbreviations

AIDS: Acquired immune deficiency syndrome
CrAg: Cryptococcal antigen
CrAg-: Cryptococcal antigen negative
CrAg+: Cryptococcal antigen positive
CT: Computed tomography
Hb: Hemoglobin
HIV: Human immunodeficiency virus
PLT: Platelet counts
WBC: White blood cell
